# Unconventional immune cells in the gut mucosal barrier: regulation by symbiotic microbiota

**DOI:** 10.1038/s12276-023-01088-9

**Published:** 2023-09-11

**Authors:** Ji-Sun Yoo, Sungwhan F. Oh

**Affiliations:** 1 Center for Experimental Therapeutics and Reperfusion Injury, Department of Anesthesiology, Perioperative and Pain Medicine, Brigham and Women’s Hospital, Boston, MA USA; 2 Graduate Program in Immunology, Harvard Medical School, Boston, MA USA

**Keywords:** Mucosal immunology, Infection

## Abstract

The mammalian gut is the most densely colonized organ by microbial species, which are in constant contact with the host throughout life. Hosts have developed multifaceted cellular and molecular mechanisms to distinguish and respond to benign and pathogenic bacteria. In addition to relatively well-characterized innate and adaptive immune cells, a growing body of evidence shows additional important players in gut mucosal immunity. Among them, unconventional immune cells, including innate lymphoid cells (ILCs) and unconventional T cells, are essential for maintaining homeostasis. These cells rapidly respond to bacterial signals and bridge the innate immunity and adaptive immunity in the mucosal barrier. Here, we focus on the types and roles of these immune cells in physiological and pathological conditions as prominent mechanisms by which the host immune system communicates with the gut microbiota in health and diseases.

## Bridging the gap: immunity and gut microbiota

This study highlights the essential role of microbiota in regulating the maintenance and function of ILC subsets and unconventional T cells in the gut. Microbial colonization influences cytokine production, affecting ILC function in barrier maintenance and protection against pathogens. Additionally, microbiota-derived antigens shape MAIT and iNKT cell populations, with their activity finely regulated by a combination of metabolites and local cytokines influenced by the microbiota. Understanding these interactions could lead to innovative strategies for enhancing gut health and effectively treating immune-mediated diseases.

## Introduction

### The intestinal mucosal surface: a dynamic hub of interaction

The mammalian gastrointestinal tract is a long and complex structure that serves as a central site where the host, microbiota, and environmental factors come together in close proximity^1^. This unique environment provides a favorable habitat for a diverse array of microbes, the majority of which coexist innocuously with the host, earning them the term “commensal” species. While these resident microorganisms do not harm the host and actively benefit it, occasional pathogen invasions pose a constant threat^2^. Therefore, to maintain a healthy state, the host has developed sophisticated mechanisms to maximize the benefits and minimize the potential risks of the microbiota.

### Noncanonical immunity in the mucosal layer: beyond typical pathogens

Traditional concepts of the immune response revolve around the presence of specific pathogens that invade the barrier and cause infection. In such cases, the goal of the immune system is to neutralize the pathogens and maintain tissue sterility. This is achieved through the selective recognition of specific molecular structures, such as pathogen-associated molecular patterns (PAMPs) by innate immune cells or specific antigens by adaptive leukocytes.

In contrast, the gut is already densely populated with a diverse microbial community. The microbiota continuously interacts with the intestinal mucosal barrier and influences host physiology, including immune functions and metabolism^2^. For example, the microbiota provides immunological benefits to the host by generating colonization resistance to invasive pathogens. The microbiota assists the host in nutrient absorption by degrading inaccessible molecules such as dietary fibers^1^. In addition, ~20% of small molecules in human blood originate from the microbiota, including necessary metabolites such as vitamins^1^.

Recent studies using germ-free (GF) animal models have demonstrated that the noninvasive introduction of bacteria contributes to the development and homeostasis of host immunity and physiology^2^. In particular, dysbiosis of the microbial community has been implicated in diseases such as inflammatory bowel disease (IBD), diabetes, and cancer^3^. Due to the beneficial effects of the microbiota, the complete elimination of microbes from the mucosal surface would be detrimental to the host. Thus, the intestinal immune system is challenged to defend against pathogens while tolerating dietary antigens and commensal microbiota.

Considering the intestinal barrier environment, it is not surprising that the intestinal mucosa harbors an extensive population of approximately 5 ×10^10^ lymphocytes, nearly five times the total number of lymphocytes in human blood^4^. Any disruption in this delicate balance of homeostasis can lead to pathophysiological conditions such as infection, inflammation, and autoimmune diseases. Therefore, specialized immune cell types are present in the mucosal layer to ensure proper immune regulation.

## Unconventional immune cells of the intestinal barrier

Recent discoveries have revealed specialized subsets of immune cells in tissues that do not fall into the traditional categories of innate or adaptive immunity^5,6^. The ontology of these cell types is not completely agreed upon, but they are most commonly classified into two groups: innate lymphoid cells (ILCs, including NK cells and ILC1-3) and innate-like lymphocytes (ILLs, such as B1 cells and several unconventional T-cell subsets)^6,7^. Among these, we will focus on cell types that have phenotypes comparable to those of conventional T cells (such as effector cytokine subtypes and/or T-cell receptor-mediated antigen recognition). ILCs, mucosal-associated invariant T (MAIT) cells, invariant natural killer T (iNKT) cells, and γδ T cells will be the primary focus of this review. We discuss these groups of innate-like lymphocytes and examine their classification, homeostatic control, and relevance to immune disease, with a particular focus on their regulation by the gut microbiota.

### Overview

In mucosal tissues, a large proportion of ILCs and unconventional T cells are present in addition to conventional T cells. Conventional T cells recognize peptide antigens presented by polymorphic major histocompatibility complexes (MHCs) through T-cell receptors (TCRs) that possess a large diversity in their αβ chains. In contrast, ILCs do not express TCRs but mirror the functions of T cells without TCRs; thus, these cells are considered innate counterparts of T cells. Unconventional T cells, such as iNKT, MAIT, and γδ T cells, express TCRs, which classifies them as adaptive immune cells^8^. However, these unconventional T cells exhibit innate-like properties due to their immune functions mediated by TCRs with limited variability, which function similarly to innate pattern recognition receptors. Unconventional αβ T cells, including iNKT and MAIT cells, recognize glycolipids or metabolites presented to nonpolymorphic MHC class Ib molecules, with limited TCR repertoires^8^. TCRs of γδ T cells bind antigen molecules in an MHC-independent manner and show limited junctional diversity^6^. As these unconventional T cells can recognize conserved nonpeptide antigens that are not recognized by conventional T cells, they can contribute an additional layer to the immune recognition system^6^.

### Evolution and development

ILCs and γδ T cells, along with other conventional T cells, appeared ~500 million years ago and have been conserved in jawed vertebrates^9^. NKT and MAIT cells, which are relatively recent additions to the mammalian T-cell family, appeared later in evolution^10^. While unconventional T cells share common effector modules with their conventional counterparts, they possess distinct antigen recognition systems^5,8^. Innate-like lymphocytes share common characteristics, including their presence as long-lived resident cells in tissues and enrichment in barrier tissues such as the gut, lungs, and skin. They tend to differentiate into effector cells during thymic development and are seeded in peripheral tissues early in life^11,12^. They play a critical role in monitoring the tissue environment, including metabolites, the immune cytokine milieu, and self-antigens, thereby serving as tissue immune surveillance. These cells exhibit effector memory-like functions in tissues, responding promptly to various environmental cues by secreting cytokines during injury and infection^5,8^. Given their developmental characteristics, tissue residence, and rapid effector functions, these specialized lymphocytes likely evolved to provide additional layers of immune protection, particularly early in life.

## Innate lymphoid cells (ILCs)

ILCs play a critical role in maintaining barrier function by sensing the tissue environment and secreting effector cytokines. These cells are derived from a common lymphoid progenitor in the bone marrow and are engrafted into peripheral tissues during the perinatal period^13^. Unlike conventional T cells, ILCs do not depend on antigen-specific TCRs and recombination-activating genes (RAGs) for their development. Instead, they rely on cytokine signaling through the common gamma chain (γc) encoded by interleukin-2 receptor gamma (IL2RG)^5^. The development of ILCs occurs in the fetal liver and adult bone marrow and involves a genetic program regulated by a number of transcription factors (reviewed elsewhere^12^). During their development, ILCs acquire tissue-homing properties and function as rapid responders in the respective peripheral tissue in which they reside.

### Classification of ILC subsets and functional polarization

ILCs can be classified into three canonical groups with five major subsets^5^. Group 1 ILCs include natural killer (NK) cells and ILC1s. Group 2 ILCs consist of ILC2s. Group 3 ILCs comprise ILC3s and lymphoid tissue inducer (LTi) cells. The identification of ILC subsets is a relatively recent discovery, with NK and LTi cells being the only known innate lymphocytes until 2008^14,15^. While a significant proportion of NK and LTi cells can be found in the blood or lymphoid organs, the newly discovered ILC subsets are primarily found in peripheral tissues^16^.

Similar to conventional T cells, ILC subsets exhibit distinct functional polarization^17^. ILC subsets share effector modules of transcription factors and cytokines with their T-cell counterparts. NK cells mirror CD8+ cytotoxic T cells, while ILC1-3 subsets resemble CD4+ helper T cells (ILC1s resemble type 1 helper T cells (T_H_1), ILC2s resemble type 2 helper T cells (T_H_2), and ILC3s resemble type 17 helper T cells (T_H_17))^5^. The transcription factors eomesodermin (EOMES) and T-box expressed in T cells (T-BET) are master regulators in NK cells that kill virus-infected and cancer cells by expressing effector cytokines such as IFN-γ, granzyme, and perforin^5^. ILC1s, characterized by T-BET expression, produce the type 1 cytokine IFN-γ upon activation by IL-12, IL-15, and IL-18^18^. GATA binding protein 3 (GATA3)- and retinoic acid-related orphan receptor A (RORa)-expressing ILC2s produce type 2 cytokines (IL-5, IL-13, and IL-9) and the epidermal growth factor amphiregulin (AREG) in response to stimulation by IL-25, IL-33, and thymic stromal lymphopoietin (TSLP)^19^. ILC3s are a major subset in the gut and the critical subset that maintains the integrity of the intestinal barrier. Retinoic acid-related orphan receptor γ (RORγt)-expressing ILC3s produce the type-3 cytokine IL-22, which is activated by IL-23 and IL-1β. ILC3s can be further subdivided into natural cytotoxicity receptor (NCR) ILC3s and C-C motif chemokine receptor 6 (CCR6) LTi-like ILC3s based on the surface expression of NCR and CCR6. NCR+ ILC3s produce IFN-γ and GM-CSF. CCR6 + ILC3s, known as LTi cells during fetal development, are crucial for the development of lymph nodes and Peyer’s patches. In adults, LTi-like ILC3s produce IL-17 and mediate MHCII antigen presentation^5^.

**Fig. 1:**
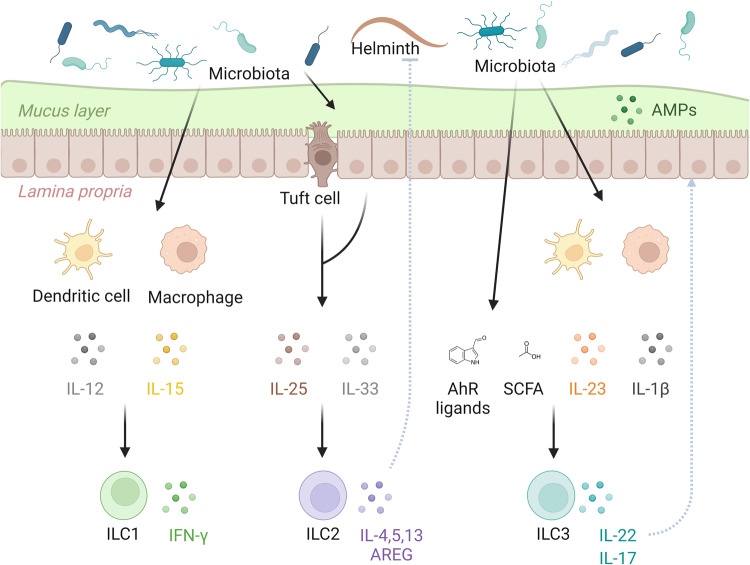
The microbiota plays a crucial role in regulating the maintenance and function of ILC subsets. Typically, microbial colonization influences cytokine production in peripheral cells such as antigen-presenting cells (APCs) and epithelial cells, thereby affecting ILC function in barrier maintenance and protection against pathogens.

Although these five major ILC subsets are commonly discussed, recent single-cell sequencing has revealed additional heterogeneity within ILC populations, showing tissue-specific phenotypes and functional plasticity (reviewed elsewhere^20^). This heterogeneity is influenced by factors such as the tissue cytokine milieu, lipid mediators, diet, hypoxia, neurotransmitters, circadian rhythm, and the microbiota^12^ (Fig. 1).

### Regulation of ILCs by the microbiota through modulation of local cytokines

#### ILC1 regulation through IL-15

Commensal bacteria influence NK cell and ILC1 function by modulating the IL-15 production of myeloid and stromal cells. For instance, bacterial colonization epigenetically regulates the production of IL-15 by mononuclear phagocytes, which is a necessary factor for NK cell priming^21^. In GF mice, mononuclear phagocytes show defects in cytokine production due to an increase in the barriers in chromatin structure, resulting in impaired NK cell priming and compromised antiviral immunity^22^. In Peyer’s patches and mesenteric lymph nodes, the microbiota can restrict ILC1s by inhibiting the secretion of IL-15 by fibroblastic reticular cells, which helps to maintain the ILC1 population^23^. The production of IL-15 by fibroblastic reticular cells is limited in a MyD88-dependent manner, suggesting that microbiota-MyD88-dependent IL-15 modulates the overactivation of ILC1s during homeostasis^23^.

#### ILC2 regulation through IL-33

Microbial colonization modulates ILC2-mediated immunity to parasites by inducing the production of IL-25 and IL-33, which are factors responsible for ILC2 activation. For example, the production of IL-25 by tuft cells is regulated by parasitic infection^24^. In particular, dietary fiber after weaning allows the colonization of *Tritrichomonas*, which produces succinate^24^. Recognition of succinate by succinate receptor 1 (SUCNR1) induces IL-25 expression in tuft cells, which subsequently activates ILC2 function in helminth immunity^25^. In addition, the microbiota influences IL-33-mediated activation of ILC2s and protection against *Clostridium difficile* infection^26^. Antibiotic-induced dysbiosis can deplete IL-33 in the gut, and fecal microbiota transplantation restores IL-33 expression^26^. A recent study showed that microbiota-derived bile acids can regulate intestinal IL-33 expression^27^. Inulin fiber increases microbiota-derived cholic acid and IL-33 expression, thereby activating ILC2-mediated type 2 inflammation^27^.

#### IL-22 produced by ILC3s

ILC3s are major subsets that produce IL-22, which plays a crucial role in maintaining barrier integrity and protecting against pathogens such as *Citrobacter rodentium*. IL-22, mainly derived from ILC3s, induces epithelial cells to secrete mucus and antimicrobial peptides, such as regenerating islet-derived protein 3 gamma (REGIIIγ) and REGIIIb, which physically separate microbes from the small intestinal epithelial surface. IL-22 also promotes the fucosylation of epithelial cell proteins through fucosyltransferase 2 (FUT2), thereby enhancing epithelial barrier function^28,29^.

#### ILC3 regulation by the microbiota

Colonization by commensal bacteria can promote IL-22 production in ILC3s by inducing the secretion of myeloid-derived IL-23. Specifically, bacterial flagellin, sensed by mononuclear phagocytes through TLR5, induces IL-23 expression, which further promotes IL-22 expression in ILC3s. Of note, the circadian clock influences the microbiota-APC-IL-23-ILC3s-IL-22 module^30^. Feeding rhythms in accordance with the circadian clock drive segmented filamentous bacteria (SFB) attachment, which are sensed by myeloid cells in a MyD88-dependent manner, leading to rhythmic IL-23 production. Rhythmic IL-23 expression induces rhythmic IL-22 expression in ILC3s, which, in turn, promotes STAT3-dependent rhythmic antimicrobial peptide expression by epithelial cells^30^. In addition to IL-22, the microbiota leads to ILC3 expression of IL-2, which maintains regulatory T (Treg) cell populations^31^. Sensing commensal bacteria by macrophages through MyD88 and Nod2 induces the production of IL-1β, which, in turn, induces IL-2 expression in ILC3s^31^. Bacterial ligands can modulate ILC3 functions, such as effector cytokine production as well as cell motility. In vivo imaging has shown that flagellin can activate the patrolling behavior of ILC3s and prevent intestinal epithelial cell death during inflammation^32^.

#### ILC3 establishment modulated by the microbiota

While the development of ILC1-2 subsets is considered to be independent of the microbiota, ILC3 development is affected by the microbiota^33^. The microbiota regulates the development of LTi-like CCR6+ and NCR+ ILC3s through epithelial cells. GF- or antibiotic-treated mice exhibit reduced NCR + ILC3 numbers in the intestine. Important factors involved in the reduction of NCR+ ILC3s include aryl hydrocarbon receptor (AhR) and RORγ (discussed in the next section). In addition, the microbiota stimulates epithelial cells to produce IL-7, which drives the IL-7-dependent stabilization of RORγt expression in ILC3s^34,35^. Moreover, NOD1 in epithelial cells recognizes muropeptides from commensal bacteria and promotes the expression of CCR6 ligands^36^. Epithelial cell-derived CCR6 ligands support the development of CCR6 + ILC3s, which are involved in the formation of isolated lymphoid follicles^36^.

### Direct recognition of the microbiota or microbiota-derived metabolites by ILCs

#### NCR (natural cytotoxicity receptor)

Several studies have reported that NCRs on NK cells can directly recognize microbial molecules. Both murine and human NCR1 (NKp46) have been shown to bind fungal adhesins of *Candida glabrata*, which is critical for controlling *C. glabrata* infection^37^. In addition, NCR1 on NK cells can bind *Fusobacterium nucleatum*, leading to the secretion of TNF-a by NK cells^38^. Human NCR2 (NKp44) and NCR3 (NKp30) have also been found to bind bacteria or fungi^39^. For example, NCR2 can bind several *Mycobacterium* species, and NCR3 recognizes *Cryptococcus* and *Candida*, promoting perforin release from NK cells^40^. Although these findings demonstrate that NCRs on NK cells can recognize pathogenic fungi and bacteria, the recognition of commensal bacteria has not been well characterized.

#### TLR (Toll-like receptor)

Activation of ILCs by TLR ligands has been reported primarily in human cells. TLR2-5 ligands can induce IFN-γ secretion and cytotoxic activity in human NK cells^36^. In the presence of IL-12 or IL-8, TLR3/9 ligands stimulate cytolytic activity and the release of IFN-γ and TNF-a in human NK cells^41,42^. Murine NK cells can be activated by TLR2 ligands in the presence of IL-2 and IFN-a during vaccinia virus infection^43^. Human LTi-like ILCs can secrete IL-5, IL-13, and IL-22 in an NF-kB-dependent manner upon stimulation with TLR2 ligands^44^. However, it should be noted that most of the knowledge about TLR-mediated activation of ILCs comes from in vitro experiments, and the functional impact of TLRs on ILCs in vivo is still limited.

#### AhR (aryl hydrocarbon receptor)

AhR ligands play a crucial role in regulating the population and function of ILCs. ILC3s express AhR and require AhR signaling for their maintenance. Loss of AhR leads to increased apoptosis of ILC3s and impairs the development of postnatal isolated lymphoid follicles, rendering mice susceptible to *Citrobacter rodentium* infection^45,46^. AhR signaling is also essential for the robust expression of IL-22 by ILC3s^46^. AhR in ILC3s interacts with RORγt and promotes AHR binding to the *Il22* locus. In contrast, AhR inhibits the function of ILC2s by suppressing the expression of interleukin 1 receptor-like 1 (IL1RL1 or IL-33 receptor)^47^.

Several dietary or microbial AhR ligands, such as hydrolytic glucosinolate and tryptophan metabolites, have been identified as modulators of ILC functions. For example, hydrolytic glucosinolate indole-3-carbinol, a well-known phytochemical from the *Brassicaceae* family of vegetables, can regulate the number of ILC3s^48^. Tryptophan metabolites such as indole-3-aldehyde produced by *Lactobacillus reuteri* promote IL-22 expression in ILC3s in an AhR-dependent manner^49^. This robust expression of IL-22 induced by *L. reuteri-*derived AhR ligands protects against *Candida albicans* infection^49^.

#### Retinoic acids

ILC subsets express retinoic acid receptor-related orphan receptors; thus, retinoic acids can affect ILC development and function. Maternal dietary retinoic acids control the differentiation of fetal RORγt + LTi cells, which are essential for the development of embryonic lymphoid organs^50^. Postnatal dietary retinoic acids also regulate ILCs, as vitamin A deficiency causes decreased ILC3s and impaired postnatal intestinal lymphoid tissues^51^. In contrast, vitamin A deficiency results in the expansion of ILC2s and enhanced immunity to helminth infections^52^. Retinoic acids induce gut-homing receptors in ILC1s and ILC3s but not in ILC2s^53^. While the regulation of ILCs by dietary retinoic acids has been extensively studied, the role of microbiota-mediated retinoic acid signaling is not well understood, although some evidence suggests that certain Clostridia species can affect retinoic acid synthesis in intestinal epithelial cells^54^.

#### Short-chain fatty acids (SCFAs)

Microbiota-derived SCFAs, including acetate, propionate, and butyrate, can enhance ILC proliferation and IL-22 production^55^. ILCs express G protein-coupled receptors (GPCRs), such as free fatty acid receptor 2 (FFAR2) and FFAR3, which sense SCFAs and activate signaling pathways, such as the PI3K-AKT-mTOR and STAT3 pathways^56^. This sensing of SCFAs by GPCRs promotes ILC proliferation and IL-22 production in the gut. SCFAs also induce the expression of the IL-1 receptor in ILC3s, increasing their responsiveness to IL-1β and enhancing IL-22 secretion^55,57^. Consequently, SCFA-mediated activation of ILC3s enhances the immune response against infections caused by pathogens such as *C. rodentium* and *C. difficile*^55,57^.

#### Antigen presentation

Some intestinal ILC subsets express MHCII and present antigens to T cells. Recent studies have shown that MHCII-expressing ILCs, likely in coordination with other MHCII-expressing cells, mediate antigen presentation to naïve CD4 T cells, promoting the development of regulatory T cells (Tregs) and ensuring immune tolerance to the microbiota^58^. These findings are consistent with the observations of dysregulation of ILC3-MHCII interactions and the reduction in ILC3 function in patients with IBD^59^. Notably, ILC3-specific MHCII-deficient mice develop invasive colorectal cancer and show resistance to anti-PD-1 immunotherapy, highlighting the importance of ILC3-mediated antigen presentation in colorectal cancer and immunotherapy responsiveness^60^. In addition, some ILC3s express CD1d, allowing for the presentation of CD1d ligands to iNKT cells, which promotes IL-22 production by ILC3s through CD1d-mediated activation^61^. Taken together, antigen presentation by ILC3s is important for maintaining peripheral tolerance to the microbiota and modulating immunity.

## Innate-like (unconventional) T cells

### Characterization and development of iNKT and MAIT cells

iNKT cells and MAIT cells are two well-characterized innate-like T-cell populations that recognize nonpeptide antigens, such as vitamins and glycolipids. iNKT cells recognize glycolipids presented on CD1d molecules, and MAIT cells recognize vitamin derivatives presented on MR1 molecules^8^. In the mouse gut, iNKT cells constitute ~1% of T cells, while MAIT cells represent less than 1% of T cells^6^. In contrast, in the human gut, iNKT cell numbers vary widely among individuals, while MAIT cells are an abundant subset^62^.

MAIT and iNKT cells are innate-like T cells that share several common characteristics. First, they recognize nonpeptide antigens presented on nonpolymorphic MHC-like molecules such as CD1d and MR1^8^. Second, both iNKT and MAIT cells exhibit a limited diversity of TCRs^8^. In mice, iNKT cells express Va14-Ja18 with Vβ8.2/Vβ7/Vβ2, whereas in humans, they express Va24-Ja18 with Vβ11. MAIT cells in mice express Va19-Ja33 with Vβ8 or Vβ6, while in humans, they express Va7.2-Ja33 with Vβ2 or Vβ13^8^. Third, iNKT and MAIT cells develop in the thymus and migrate to peripheral tissues after birth^6^. During thymic development, the selection process for iNKT and MAIT cells differs from that of conventional T cells. Conventional T cells originate from CD4 CD8 double-positive (DP) thymocytes selected by thymic epithelial cells. iNKT and MAIT cells are generated from DP thymocytes selected by other DP thymocytes^63^. The sequential rearrangement of TCR sequences, with Va14 and Va19 located distally in the TCRa locus, makes the half-life of DP thymocytes critical for the development of iNKT and MAIT cells^63^. Fourth, the abundance of iNKT and MAIT cells is imprinted at an early age around the weaning period^11^. Finally, the abundance of iNKT and MAIT cells in tissues is strongly influenced by microbiota colonization^64,65^.

Like ILCs, iNKT and MAIT cells can reflect some effector T-cell subsets and rapidly produce cytokines upon activation^6^. These cells provide immunity against various bacterial and viral infections and play roles in cancer and other immune-related diseases, such as allergy, type 1 diabetes, and IBD^6^.

### Characterization and development of γδ T cells

γδ T cells are evolutionarily conserved immune cells present in all animals with an adaptive immune system^6,66^. Multiple subsets of γδ T cells have been reported to recognize a variety of ligands, although the precise mechanisms of antigen recognition by γδ T cells are still not fully understood^66^. In the gut, γδ T cells contribute to maintaining intestinal barrier function by promoting intestinal epithelial cell proliferation and antimicrobial peptide production^67^. Consequently, TCRδ-knockout mice show increased susceptibility to colitis induced by dextran sulfate sodium (DSS) or 2,4,6-trinitrobenzene sulfonic acid (TNBS)^67^.

Several subsets of γδ T cells are found in the gut, including γδ intraepithelial lymphocytes (IELs) and lamina propria γδ17 T cells^67^. These subsets exhibit distinct effector functions that are shaped by environmental factors, including food and microbes^67^. γδ IELs are motile cells involved in tissue surveillance and the maintenance of barrier integrity^67^. They also produce the growth factor KGF1 (keratinocyte growth factor 1), which promotes the proliferation of intestinal epithelial cells^67^. Lamina propria γδ17 T cells, on the other hand, produce type-3 cytokines such as IL-17 and share functional characteristics with Th17 cells^67^.

Recent studies have identified specific interactions between γδ T-cell TCRs and butyrophilins (BTNs), which belong to the immunoglobulin superfamily and share sequence similarity with the B7 family of costimulatory receptors^68^. Human Vγ4 + IELs bind to BTNL3–BTNL8 heterodimers, whereas murine Vγ7 + IELs bind to BTNL1–BTNL6 heterodimers expressed on intestinal epithelial cells^66^. These γδ IELs, characterized by CD8αα expression, represent the predominant subset of γδ T cells in the intestine, constituting ~50–60% of IELs in mice and 10-20% of IELs in humans^67^. In mice, Vγ7 + γδ IELs develop in the thymus, home to the gut during the perinatal period, and undergo extrathymic selection in the gut at the weaning stage^67^.

### Regulation of innate-like T cells by the microbiota

**Fig. 2:**
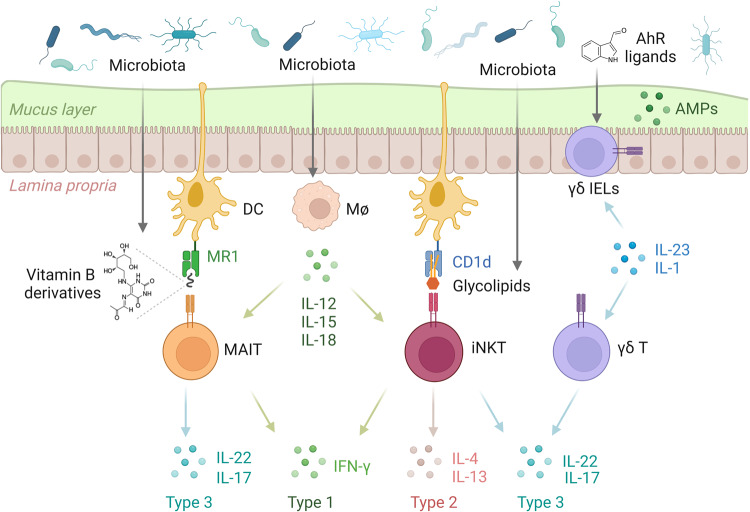
The microbiota affects the development and function of innate-like T cells in the gut. MAIT and iNKT cell populations are shaped by the presence of microbiota-derived antigens. The activity of innate-like T cells is finely regulated by a combination of metabolites and local cytokines influenced by the microbiota.

The regulation of innate-like T cells by the microbiota is a fundamental element of the immune system. These innate-like T cells play a crucial role in immune surveillance and response, particularly in mucosal barrier tissues where the microbiota influence the development, function and regulation of innate like T cells (Fig. 2).

#### iNKT cells

##### iNKT cell development

The microbiota plays a crucial role in the development and function of iNKT cells. Studies have shown that iNKT cells harvested from mice of different commercial vendors exhibit differences in frequency, Vβ7 usage, and TNF-a production^69^. The impact of the microbiota on iNKT cells varies across different tissues. In GF mice, the number of iNKT cells decreases in the liver and spleen, while it increases in the lung and colon, leading to hyperreactivity to iNKT cell-mediated colitis and allergic responses^70^. The increased number of iNKT cells in GF mice can be normalized by colonization within the first two weeks after birth^70^. In contrast, the decrease in iNKT cells in the liver and spleen of GF mice can be normalized by colonization in adult mice^70^. Mechanistically, the colonization of commensal bacteria induces epigenetic changes in the promoter of the chemokine Cxcl16, which mediates the accumulation of iNKT cells in the gut^70^. In addition, a recent study has shown that this early imprint of iNKT cell establishment in the colon is mediated by embryonic macrophages rather than bone marrow-derived macrophages^71^. GF mice exhibit elevated levels of embryonic macrophages in the colon, promoting iNKT cell expansion^71^. Depletion of macrophages in the second week of life results in a decrease in iNKT cells in the colon^71^.

##### Microbiota-derived iNKT cell antigens

The CD1d ligands originating from the microbiota directly influence the development and function of iNKT cells in the gut. Various CD1d ligands sourced from different bacteria, such as *Sphingomonas*, *Streptococcus*, and *Borrelia*, have been reported thus far^62^. Among these, *Bacteroides fragilis*, a prevalent bacterial species in the human gut, produces alpha-galactosylceramides (BfaGCs), the prominent iNKT antigen^65,72^. BfaGCs modulate iNKT cell proliferation in early life, and the colonization of *B. fragilis* can normalize the elevated number of iNKT cells in GF mice^65^. Notably, endogenous BfaGC structure^72^ is dictated by host dietary factors, which determine NKT modulatory functions.

In addition to endobiotic metabolites, secondary metabolites produced by the microbiota, such as the SCFA butyrate, can modulate the iNKT cell function of cytokine production by inhibiting histone deacetylase (HDAC)^73^. In addition to the direct regulation of iNKT cells by small molecules derived from the microbiota, commensal bacteria can modulate iNKT cell functions by activating antigen-presenting cells through recognition by TLRs and other receptors, such as Dectin-1, Rig-I, and Nod1/2^74^.

#### MAIT cells

The maintenance of MAIT cells is highly dependent on the colonization of commensal bacteria^75^. GF mice exhibit reduced numbers of MAIT cells in several tissues, including the skin, lung, intestine, liver, spleen, and thymus^75^. The development of MAIT cells is largely dependent on the specific riboflavin (vitamin B2) intermediate 5-OP-RU presented in the thymus^64^. Similar to iNKT cells, the establishment of MAIT cells is critically influenced by early-life exposure to commensal bacteria, and colonization with riboflavin-synthesizing bacteria within the first three weeks after birth can normalize the number of MAIT cells^75^. Various riboflavin derivatives derived from bacteria have been identified as MAIT cell ligands, and the structural differences in these ligands influence their binding to MR1 and subsequent MAIT cell activation^76^. Multiple groups have shown that MAIT cells recognize various bacteria and fungi, including *Escherichia coli*, *Salmonella*, *Streptococcus*, *Mycobacterium*, and yeast^62^. In addition to direct ligand recognition, the activation of MAIT cells can also occur in a TCR-independent manner by APC-derived cytokines such as IL-12 and IL-18^77^. Although TCR-independent induction of MAIT cells can occur, TCR stimulation is still required for the expression of repair-associated genes in human MAIT cells^78^.

#### γδ T cells

##### γδ17 T cells

Microbial colonization is essential for the establishment of lamina propria γδ17 T cells^67^. GF- or antibiotic-treated mice exhibit decreased numbers of γδ17 T cells in the lamina propria^79^. The sensing of microbiota by intestinal epithelial cells via MyD88-dependent mechanisms promotes the production of IL-15, which is required to maintain the γδ17 T-cell population^79^. The production of IL-17 by γδ17 T cells is also regulated by the microbiota through myeloid cell-derived IL-1 and IL-23, which promote IL-17 expression by γδ17 T cells^80^. Mechanistically, optimal IL-17 production by γδ17 T cells requires an increase in interleukin 1 receptor type 1 (IL-1R1) expression, which is induced by commensal colonization and vav guanine nucleotide exchange factor 1 (VAV1) signaling^81^. In contrast, microbiota-derived SCFAs, such as propionate, also impact γδ17 T-cell function by inhibiting HDAC activity and repressing IL-17 production^82^.

##### γδ IELs

Unlike γδ17 T cells, the establishment of γδ IELs is independent of microbial colonization, but the function of γδ IELs is subject to regulation by the local environment shaped by the microbiota^79^. For example, γδ IELs patrol between epithelial cells, and their migration is controlled by MyD88-dependent recognition of microbiota by epithelial cells^79^. Epithelial cells also produce IL-23 upon TLR-mediated recognition of microbes, which drives γδ IELs to produce IL-22. IL-22, in turn, promotes angiogenin 4 production by Paneth cells^79^. Notably, γδ IELs from GF mice have defects in lytic activity, and their cytotoxicity is dependent on exposure to the gut microbiota^79^. In addition, AhR signaling has been identified as crucial for maintaining the gut γδ IEL population, although the exact microbial contributions to the AhR-γδ IEL circuit remain unknown^83^.

## Unconventional immune cells and immune-related diseases

### Infectious diseases

Innate-like lymphocytes play a critical role in the defense against various pathogens. These cells mount an immune response by secreting effector cytokines during the early stages of infection. Some innate-like lymphocytes have cytotoxic activity and can kill infected cells directly, while others activate neutrophils and phagocytes by producing IL-17 and IFN-γ^6^. These cells are involved in defense against viral infections (e.g., Rotavirus, Norovirus, and Cytomegalovirus), bacterial infections (e.g., *Citrobacter*, *Yersinia*, *Salmonella typhimurium*, *Mycobacterium*, *Escherichia*, *Listeria*, and *Clostridium difficile*), fungal infections (e.g., *Candida albicans*), and parasitic infections (e.g., *Nippostrongylus*, *Heligmosomoides*, *Trichinella*, *Strongyloides*, *Trichuris*, *Toxoplasma*, and *Giardia*)^84^. Notably, human subjects bearing MR1 point mutations that result in the absence of MAIT cells are susceptible to bacterial and viral infections^85^. In addition to their role in preventing infections, innate-like lymphocytes also contribute to enhancing barrier function and have been implicated in tissue repair processes^5^. For example, ILC2s produce IL-13 and amphiregulin, which are required for intestinal epithelial regeneration, crypt stem cell proliferation, and mucin production^84^. ILC3s produce GM-CSF, which contributes to wound healing, and IL-22, which maintains crypt stem cells and aids in tissue repair^84^.

### IBDs

Dysregulated innate lymphocytes have been implicated in several inflammatory diseases, including celiac disease, diabetes, and IBDs. Notably, several studies have reported an association between innate-like lymphocytes and IBD in affected patients. Specifically, the accumulation of ILC1s has been observed in the inflamed terminal ileum of individuals with Crohn’s disease, a form of IBD^86^. Additionally, alterations in the expression of AhR in ILCs, as well as the conversion between ILC3 and ILC1 subsets, have been reported^87^. These findings suggest that the differential expression of AhR in ILCs and the conversion between ILC subsets may have clinical relevance in IBD patients.

Notably, a recent study demonstrated an enrichment of a specific subset of innate-like T cells in the blood of individuals diagnosed with Crohn’s disease^88^. This distinctive group of T cells is characterized by their possession of semi-invariant TCR alpha chains and display an innate-like phenotype, as evidenced by their specific gene expression patterns^88^. While their existence and enrichment have been established, further investigation is essential to unravel the intricacies of their immunophenotype and antigen reactivity. Such investigations will undoubtedly contribute to our deeper understanding of the functional characteristics and potential implications of these innate-like T cells in Crohn’s disease.

### Cancers

ILC1-3 subsets can be context-dependent anti- or protumorigenic cells^12,89^. ILC2 levels are increased in the circulating blood of gastric cancer patients, but IL-5 and IL-9 of ILC2s show antitumorigenic effects^90^. High levels of IL-23 have been reported in human and murine colon cancer, and IL-23 may promote tumor growth^89,91^. Consistent with these findings, IL-23R KO mice become resistant to B16F10 melanoma tumor growth and spontaneous development of colon cancer^91,92^. In contrast, several studies have reported a protective role for ILC1s and ILC3s in cancer. ILC1s exhibit potent cytotoxic activity against cancer cells^93^. IL-22 can mediate the protection of intestinal stem cells against carcinogens that induce DNA damage in homeostasis^94^. One study highlights the significance of ILC3-mediated antigen presentation via MHCII in both colorectal cancer development and the efficacy of immunotherapy^60^. Of note, *Lactobacillus reuteri* and SCFAs were reduced in the hepatocellular carcinoma mouse intestine^95^. Fecal transplantation from specific pathogen-free mice or administration of *L. reuteri* elevates acetate levels and promotes an antitumor effect^95^. Moreover, the combination of acetate with PD1 blockade therapy significantly enhanced antitumor immunity^95^. In accordance with this finding, acetate and hepatic ILC3 infiltration were negatively correlated in individuals with hepatocellular carcinoma^95^.

In addition to ILCs, several unconventional T-cell subsets, including iNKT and MAIT cells, are recognized for their antitumor activities^96^. Although there are numerous clinical studies targeting unconventional T-cell immunotherapy (reviewed elsewhere^96,97^), the interplay between unconventional T cells and the microbiota in the context of cancer has not been fully elucidated.

## Conclusion and future perspectives

Pioneering work by several groups on innate leukocytes has led to a consensus that innate-like lymphocytes play an important role in the development, homeostasis, and regulation of the gut mucosal immune system as well as in various diseases. Furthermore, the influence of the microbiota on the regulation of innate lymphocytes is clear. However, due to their complex nature, the specific mechanisms by which microbiota-derived molecules regulate these cells have remained largely elusive. To gain mechanistic insights, reductionist tools such as the use of gnotobiotic (GF or defined microbiota-associated) animals or tissue organoids can be employed. In parallel, in addition to the major human diseases mentioned above, the functions of innate-like lymphocytes have been implicated in several types of autoimmune diseases.

Moreover, there’s a growing recognition of the association of gut microbiota dysbiosis with autoimmune diseases. These findings emphasize the potential link between dysregulated microbiota and innate-like lymphocytes in autoimmune diseases. Further investigation into the role of innate-like lymphocytes in autoimmune diseases may open new avenues for therapeutic intervention. Probiotics, prebiotics, and fecal microbiota transplantation are promising strategies that could be used to manipulate gut microbiota and influence innate-like lymphocyte activity. By further exploring the specific mechanisms underlying these interactions and leveraging the gut microbiota as a therapeutic target, innovative strategies may be developed to enhance gut health and effectively treat immune-mediated diseases.
